# Elimination of hepatitis C virus in Germany: modelling the cost-effectiveness of HCV screening strategies

**DOI:** 10.1186/s12879-019-4524-z

**Published:** 2019-12-02

**Authors:** Christian Krauth, Siegbert Rossol, Gustaf Ortsäter, Achim Kautz, Kathrin Krüger, Babette Herder, Jona Theodor Stahmeyer

**Affiliations:** 1 Institute for Epidemiology, Social Medicine and Health Systems Research, Hannover Medical School, Carl-Neuberg-Str. 1, D-30625 Hannover, Germany; 2 Department of Internal Medicine, Krankenhaus Nordwest, Frankfurt am Main, Germany; 3 Quantify Research, Stockholm, Sweden; 4 Liver Help Project Ltd, Cologne, Germany

**Keywords:** Hepatitis C virus, Screening, Markov model, Cost-effectiveness

## Abstract

**Background:**

Chronic hepatitis C is a major public health burden. With new interferon-free direct-acting agents (showing sustained viral response rates of more than 98%), elimination of HCV seems feasible for the first time. However, as HCV infection often remains undiagnosed, screening is crucial for improving health outcomes of HCV-patients. Our aim was to assess the long-term cost-effectiveness of a nationwide screening strategy in Germany.

**Methods:**

We used a Markov cohort model to simulate disease progression and examine long-term population outcomes, HCV associated costs and cost-effectiveness of HCV screening. The model divides the total population into three subpopulations: general population (GEP), people who inject drugs (PWID) and HIV-infected men who have sex with men (MSM), with total infection numbers being highest in GEP, but new infections occurring only in PWIDs and MSM. The model compares four alternative screening strategies (no/basic/advanced/total screening) differing in participation and treatment rates.

**Results:**

Total number of HCV-infected patients declined from 275,000 in 2015 to between 125,000 (no screening) and 14,000 (total screening) in 2040. Similarly, lost quality adjusted life years (QALYs) were 320,000 QALYs lower, while costs were 2.4 billion EUR higher in total screening compared to no screening. While incremental cost-effectiveness ratio (ICER) increased sharply in GEP and MSM with more comprehensive strategies (30,000 EUR per QALY for total vs. advanced screening), ICER decreased in PWIDs (30 EUR per QALY for total vs. advanced screening).

**Conclusions:**

Screening is key to have an efficient decline of the HCV-infected population in Germany. Recommendation for an overall population screening is to screen the total PWID subpopulation, and to apply less comprehensive advanced screening for MSM and GEP.

## Background

Chronic hepatitis C is a global public health burden. More than 185 million people have been infected with the hepatitis C virus (HCV) worldwide and approximately 350,000 patients die each year from HCV-related diseases [1]. Estimates assume that about 27% of liver cirrhosis and 25% of hepatocellular carcinoma are attributable to chronic HCV [2]. Data from the German National Health and Examination Survey (DEGS1) show an anti HCV-prevalence of 0.3% in Germany [3]. Considering a higher prevalence in risk-groups such as drug abusers, recent studies estimate the number of infected people at 275,000 [4]. The majority of the patients are infected with HCV genotype 1 or 3 [5]. European data show an anti HCV-prevalence of about 1.1%, ranging from 0.1% in Belgium, Ireland and the Netherlands to 5.9% in Italy, with an estimated total of 5.6 million HCV cases [6]. A large part of infected patients are unaware of their disease and most infections remain undiagnosed until serious complications such as liver cirrhosis and hepatocellular carcinoma occur [7].

The historical dual therapy with pegylated interferon and ribavirin was the standard of care for more than a decade until first generation protease inhibitors telaprevir and boceprevir were approved for the treatment of patients with HCV genotype 1 in 2011. In 2014 the treatment of HCV has significantly improved with the introduction of the first all-oral direct-acting antiviral drug (sofosbuvir). Other direct acting antivirals (DAAs) simeprevir, daclatasvir, sofosbuvir/ledipasvir, ombitasvir/paritaprevir/ritonavir plus dasabuvir, elbasvir/grazoprevir, and pangenotypic effective combinations sofosbuvir/velpatasvir, sofosbuvir/velpatasvir/voxilaprevir, and glecaprevir/pibrentasvir subsequently followed, ultimately leading to interferon-free strategies. Meanwhile DAAs achieve sustained viral response (SVR) rates of > 98%, show minimal adverse effects and shorten treatment duration considerably [8].

Thus, for the first time elimination of hepatitis C virus seems feasible. As HCV infection often remains undiagnosed, screening and early diagnosis are crucial for improving health outcomes of HCV patients. The World Health Organization pursues the ambitious target to eliminate HCV until 2030 [9]. However, as the new regimens are still expensive, effectiveness and cost-effectiveness of screening (and subsequent treatment) should be assessed. Whereas some European countries have implemented different approaches and are on track to achieve the goal of HCV elimination until 2030, other countries like Germany have not yet implemented a nationwide strategy to reduce the burden of HCV and to achieve the goal of HCV elimination [10].

The aim of the present study was to analyze the impact of different nationwide screening strategies (for different risk groups and the total population) on the long-term development of HCV prevalence, liver-related mortality, quality-adjusted life years (QALYs), and healthcare costs, and to identify the most cost-effective screening strategy (among those analyzed). We used Germany as an example for high income countries.

## Methods

**Fig. 1:**
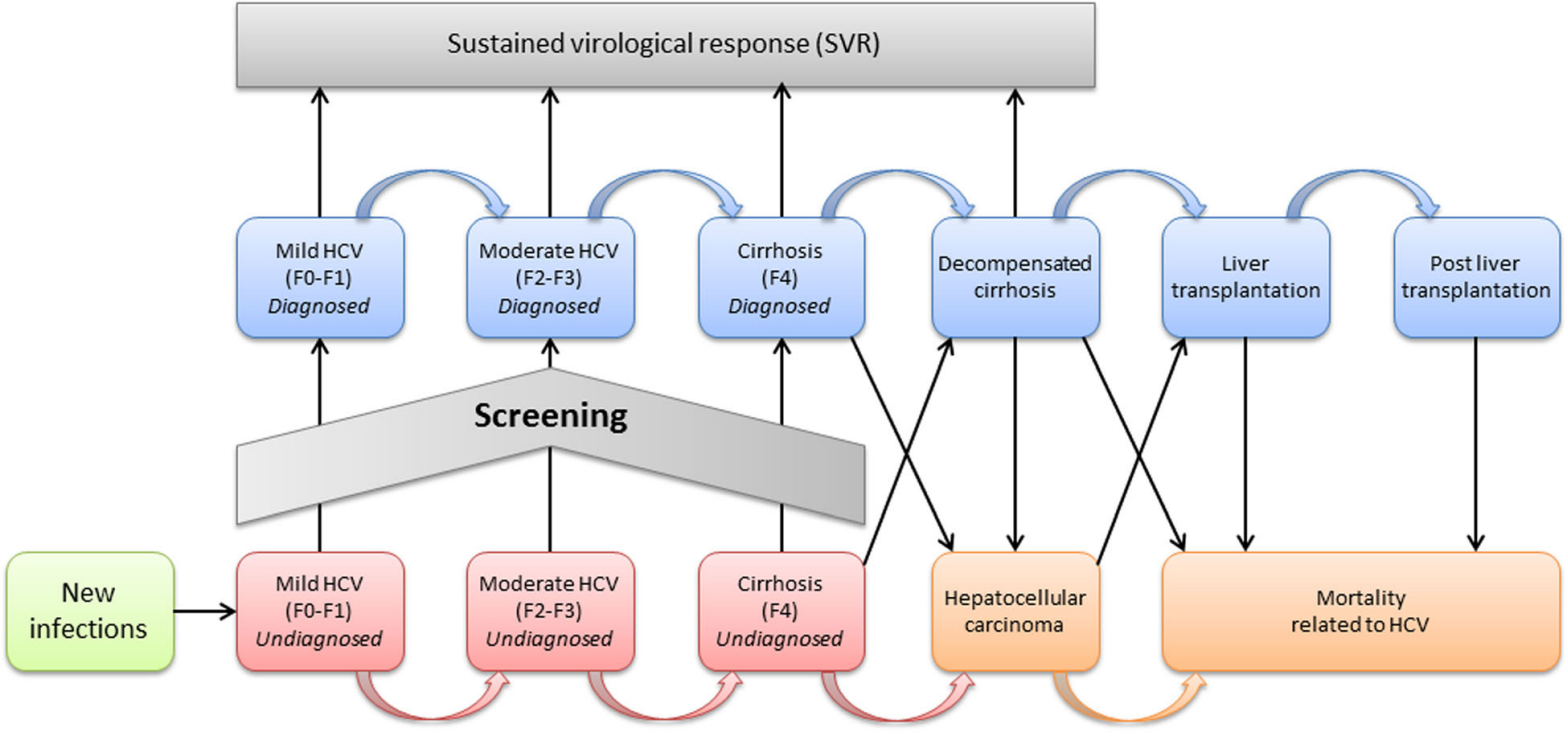
Model structure

We applied a sequential, multi-cohort, health-state transition (Markov) model to examine population outcomes and cost-effectiveness of HCV screening over a 25-year time horizon (from 2015 to 2040). In contrast to the WHO target to eliminate HCV until 2030, the time horizon of this model is enhanced to 2040 as elimination of HCV until 2030 seems to be unrealistic given the lack of a nationwide organized strategy to eliminate HCV. Further, we want to illustrate the development of the HCV induced burden on the long term if no activities are implemented. Model structure and assumptions are summarized in Fig. 1. The model was programmed with Microsoft Excel.

### Model populations

The model considers three distinct subpopulations as main target groups for HCV screening: general population (GEP), people who inject drugs (PWID) and HIV-infected men who have sex with men (MSM). The subpopulations differ in the transmission of the HCV infection.

The GEP population (i.e. the total German population except for PWID and MSM) includes persons with increased risks for HCV infection, like immigrants from Mediterranean or East European countries, recipients of HCV infected blood transfusions prior to 1992 when universal blood product screening was introduced [5, 11] or healthcare workers who were exposed to infected blood (e.g. via needle stick injuries). We estimated that the GEP population includes about 70% (or 191,500) of all HCV-infected persons (275,000) [4], but is assumed not to experience ongoing transmission risks. Infected GEP population is mainly aged 35+ and about 48% of infected persons are aware of their HCV infection (and need not be screened) [11].

In the PWID population HCV infection is transmitted via common use of injecting equipment. The PWID population consists of two subgroups: PWIDs in community who regularly inject drugs (PWID-C) and (former) PWIDs undergoing substitution therapy (PWID-S). Screening measures are easier implemented in PWID-S as they are in regular contact with the healthcare system, while PWID-C should be approached by the community (i.e. low-threshold screening measures executed by non-medical personnel). At model start about 90% of assumed annual new infections (4300 in 2015) [12] are supposed to occur in PWIDs and prevalence rate (42%) is very high (implying 79,800 HCV-infected PWIDs) [13]. It is assumed that all PWIDs need to be screened, though a high percentage of infected PWIDs might be aware of their HCV infection (71%), as main efforts in screening PWIDs are needed to get them into the (screen and treat) program.

HCV infection in MSM is transmitted via sexual contacts. The MSM subpopulation is a small group within the HCV-infected population (3700), but represents about 10% of all incident cases and faces an increasing prevalence rate over the last years (assumed to be about 7% at model start) [14, 15]. Similar to PWIDs, it is assumed that all MSM need to be screened. Information on population size, prevalence and awareness rates are summarized in Additional file 1: Table S1.

### Model structure

The model reflects the natural course of the HCV infection (see Fig. 1). Infected patients progress through various levels of fibrosis (measured by Metavir score), decompensated cirrhosis, hepatocellular carcinoma, liver transplantation and death with transition rates derived from the literature [16, 17] (see Additional file 1: Table S1.

Treatment of HCV-infected patients is performed with most advanced DAAs, as being used in German clinical practice. Non-cirrhotic patients (F0-F2) who achieve sustained viral response (SVR) are considered cured and their liver damage is assumed to resolve. If re-infected, patients re-enter the infected population with an undamaged liver. Patients with failed treatment remain infected and their liver disease continues to progress. In patients with Metavir score F3+ liver damage is assumed to remain and continues to progress, even if SVR is achieved, though more slowly than in patients with active HCV infection [18].

All model parameters are based on published literature (see Additional file 1: Table S1.). SVR rates are derived from current clinical trials [8, 19]. Prevalence (stratified by age and disease severity) and incidence rates, distributions of genotypes, disease stages and transition probabilities, and age groups among the infected populations are based on estimates from the literature [4]. All-cause mortality rates are derived from German life tables [20].

### Screening scenarios

Our model compares four alternative one-time screening strategies: (1) no screening, (2) basic screening, (3) advanced screening and (4) total screening which differ by the number of detected (and treated) HCV-infected persons. Screening approaches (and percentage of diagnosed and treated patients) differ between GEP, PWID and MSM subpopulations. Maximum HCV treatment capacity is supposed to be 25,000 patients per annum (as was the actual number of treatments in 2015) [12]. All treatment eligible patients have an equal chance to get HCV treatment (irrespective of Metavir score).

#### No screening

The no screening scenario represents the current situation in Germany. At model start, the total number of new diagnoses is about 4900 [12]. If no screening is executed, the number of new diagnoses in GEP is assumed to equate the number of new F4 cases (550 at model start) (as at this disease stage patients notice severe symptoms of their disease and will contact the healthcare system), while in PWIDs and MSM new diagnoses are supposed to equate incidence numbers (about 3900 and 430 cases, respectively). In the no screening scenario we assume that the current treatment policy is continued in the future. Treatment numbers in PWID and MSM subpopulations equate incidence numbers as we assume long-term stable numbers in the PWID and MSM subpopulations if no screening is executed.

#### Basic screening

In the GEP subpopulation, basic screening is applied to persons who participate in check-up 35+, a biannual free of charge medical check-up in Germany for people with age above 35 years. In basic screening a three-stage test is applied: (1) risk-based questionnaire and ALT test (for all check-up 35+ participants), (2) anti-HCV test (for all participants with increased risk or abnormal ALT values) and (3) HCV-RNA test (for all individuals with positive anti-HCV test). 48% of all eligible persons (about 45 million) participate in check-up 35+ [21], 53% of check-up participants need further anti-HCV-testing and participants with HCV-antibodies (about 0.4% prevalence) need HCV-RNA testing (see Additional file 1: Table S2). This three-stage approach identifies about 85% of all infected check-up 35+ participants [11]. In the PWID subpopulation basic screening is restricted to PWID-S (undergoing substitution therapy) with an assumed 50% participation rate as it is assumed for the MSM subpopulation, too. In both, PWID-S and MSM a two-stage screening test (anti-HCV test and RNA test) is applied as recommended by national and international guidelines [8, 22, 23].

#### Advanced screening

Advanced screening differs from basic screening as follows: the risk-based screening in the GEP population is expanded to the total general population aged 16+ years (considering lower prevalence in people aged 16 to 34 years) with a supposed 80% participation rate (assuming that additional physician fees accrue for including patients into the screening program). In the PWID subpopulation screening is broadened to PWID-C (assuming 40% participation rate among PWID-C and 80% among PWID-S and MSM). Extra costs for social workers approaching PWID-C individuals are considered.

#### Total screening

The total-screening scenario assumes that the entire population is screened and all HCV-infected persons are treated in the subsequent years after diagnosis. A two-stage screening test for all individuals is applied. Increasing costs for including individuals into the screening program (similar to the advanced screening scenario) are considered.

#### Mixed screening

Depending on cost-effectiveness results derived from the screening model the optimal screening strategy might differ between subpopulations. Thus, an overall optimum might combine e.g. basic screening in general population, advanced screening in PWIDs and total screening in MSM (or other combinations).

### Outcomes

To analyze long-term effectiveness and cost-effectiveness we calculate long-term development of HCV prevalence, liver-related mortality and lost quality-adjusted life years (QALYs) (due to HCV-infection and compared to non-infected persons) and derive incremental cost-effectiveness ratios (ICERs) for the screening strategies analyzed.

Health state utilities reflect the quality of life of HCV-infected patients in each health state on a scale from 1 (perfect health) to 0 (death). Information is derived from German studies which used EuroQoL-5D for determination of quality of life in HCV infected and non-infected persons [24, 25]. If no information is available for certain therapies, data from similar treatments are used as an approximation (see Additional file 1: Table S1).

When conducting cost-effectiveness analyses a threshold at which health services could be regarded as *“cost-effective”* has to be set. In Germany there is no official cost-effectiveness threshold and cost-effectiveness plays a minor role in the decision if health services are implemented. For this analysis we selected a fictive threshold of 20,000 EUR per QALY which is based on the official cost-effectiveness threshold of 20,000 GBP per QALY in the UK [26].

### Cost data

Cost data include screening, treatment and indirect cost. Screening costs comprise costs for the (two-stage or three-stage) test and time expenditures for approaching and including specific target groups (as PWID-C). Screening cost data are derived from the German uniform physicians’ fee scale in the statutory health insurance scheme. Costs of treating hepatitis C include antiviral treatment associated costs (as pharmaceuticals and diagnostic procedures) and costs of disease progression (health state costs). Pharmaceutical treatment costs vary between different treatment options. Since the introduction of first DAAs prices have significantly decreased. We assumed average treatment costs of 34,000 EUR reflecting recent costs developments [27, 28]. An annual price reduction of 4% for the DAA was also taken into account. Costs for diagnostic procedures are adapted and updated from a published study on guideline-based treatment costs [29, 30]. Health state costs are derived from published literature [31–33]. Furthermore, HCV is associated with increased indirect cost. We consider productivity losses due to absenteeism and presenteeism and early retirement based on published studies [34–37]. Indirect cost data are derived from Federal Office of Statistics [38]. Cost data are summarized in Additional file 1: Table S1.

The study is conducted from a societal perspective. All cost data are reported in 2015 euros. An annual discount-rate of 3% is used for costs and QALYs (as recommended by the German Institute for Quality and Efficiency in Health Care [39].

### Sensitivity analyses

We performed deterministic sensitivity analyses to evaluate the robustness of our screening model and to examine the effects of parameter uncertainty on incremental cost-effectiveness ratios. We varied cost parameters, incidence and prevalence by ±25%, diagnosis rate by ±10%, SVR-rates by ±5% (as no detailed information on point estimates is available), and treatment numbers by ±5000 (to assess the impact of expanding or downsizing treatment capacities). Variation of utilities was according to 95% confidence interval, and discount rates were set at 0 and 5%. Furthermore, we examined the impact of substantial treatment price reductions (to 25,000 and 20,000 EUR).

## Results

### Comparison of screening strategies in the total population

**Fig. 2:**
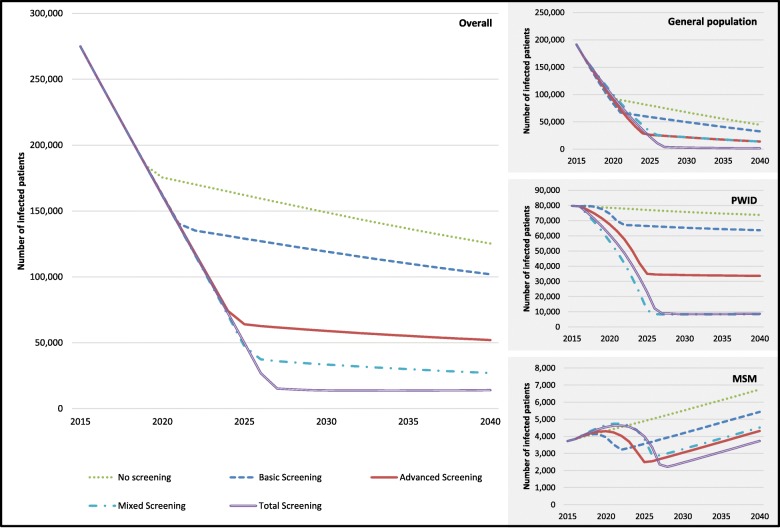
Development of infected patients: overall and in subgroups

Figure 2 shows the total HCV-infected population over time in the four screening strategies analyzed. Starting with a population of 275,000 HCV-infected persons [4] numbers are declining in all screening scenarios, but do so quite differently. As the number of detected (and treatment eligible) persons is increasing with more comprehensive screening procedures, full annual treatment capacity (of 25,000 treatments per year) is utilized until 2025 in total screening compared to 2018 in no screening (and in between in basic and advanced screening) (see Fig. 2). Thus, after 25 years (i.e. in 2040) the number of infected patients drops to between 14,000 (in total screening) and 125,000 (in no screening).

**Fig. 3:**
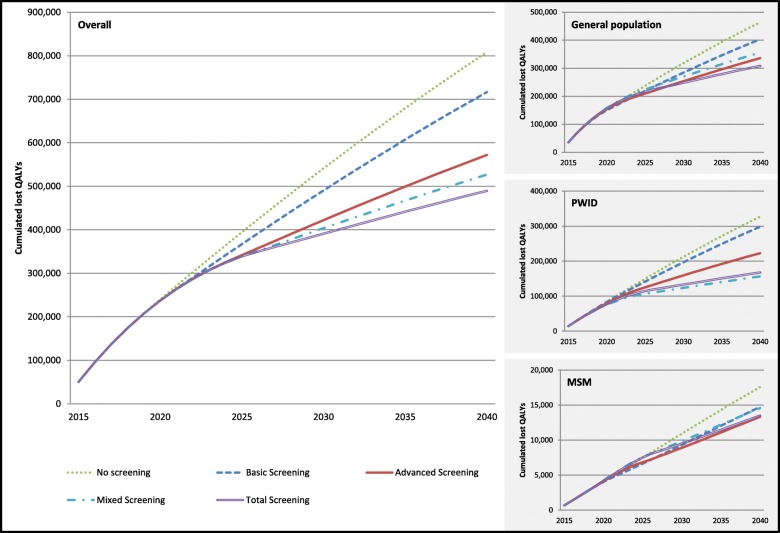
Cumulated annual lost QALYs: overall and in subgroups

Similarly, the (cumulated) number of premature (HCV-related) death is decreasing with more comprehensive screening approaches (from 37,000 in no screening to 22,000 in total screening in a 25 years period) and the (discounted) total number of lost QALYs (compared to non-infection) is decreasing as well (from about 810,000 in the no screening scenario to 490,000 in the total screening scenario) (see Fig. 3).

**Fig. 4:**
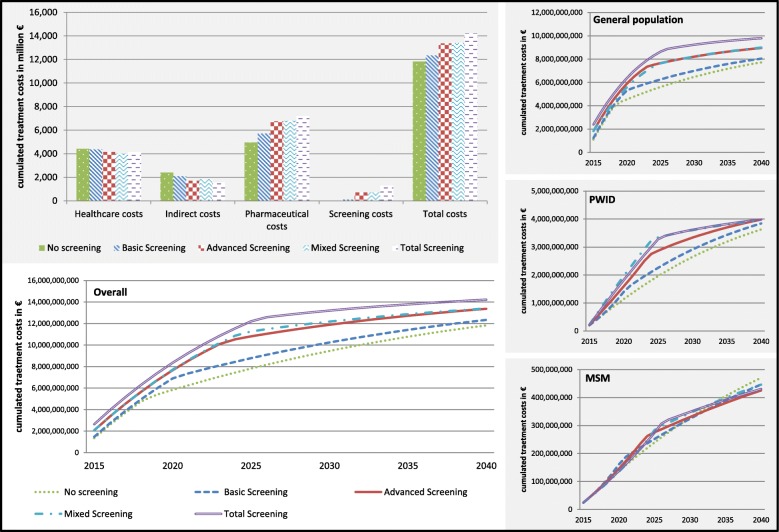
Cumulated annual costs: overall and in subgroups

The higher number of treatments in more comprehensive screening strategies is accompanied by increasing costs. Figure 4 depicts (discounted) total costs and cost components (i.e. screening cost, treatment cost, non-treatment healthcare cost, indirect cost), and shows total costs over time for each of the four screening strategies. While screening and treatment costs are increasing with more comprehensive screening strategies, non-treatment healthcare costs and indirect costs are decreasing (due to the prevention of progressive health states). Overall, total costs are increasing from 11.8 billion EUR in no screening to 14.2 billion EUR in total screening.

As shown in Table 1 incremental cost-effectiveness ratios are slightly increasing with more comprehensive screening strategies (with incremental cost-effectiveness ratios between 5500 EUR per QALY for basic versus no screening and 10,200 EUR per QALY for total versus advanced screening). Assuming a threshold of 20,000 EUR per QALY (which is adapted from National Institute of Clinical Excellence (NICE) in the UK as there is no official German threshold) total screening (i.e. the most comprehensive strategy) would be recommended to apply for a German HCV screening program if pure (i.e. either basic, advanced or total screening for all) strategies in the overall population are considered.

**Table 1:** ICERs for all screening strategies

ICER (A) vs (B)		(B)			
		No screening	Basic Screening	Advanced Screening	Mixed Screening
(A)	Total population				
	Basic Screening	5592 EUR			
	Advanced Screening	6525 EUR	7116 EUR		
	Mixed Screening	5590 EUR	5589 EUR	689 EUR	
	Total Screening	7490 EUR	8257 EUR	10,258 EUR	21,801 EUR
	Subgroups				
	General population				
	Basic Screening	5322 EUR			
	Advanced Screening	9635 EUR	13,481 EUR		
	Total Screening	13,317 EUR	18,379 EUR	30,370 EUR	
	PWID				
	Basic Screening	7548 EUR			
	Advanced Screening	3406 EUR	1838 EUR		
	Total Screening	2240 EUR	1077 EUR	30 EUR	
	MSM				
	Basic Screening	− 8445 EUR^a^			
	Advanced Screening	−10,614 EUR^a^	−14,962 EUR^a^		
	Total Screening	− 9796 EUR^a^	−12,898 EUR^a^	dominated^b^	

^a^ (A) dominates (B): less lost QALYs and lower costs in (A) compared to (B)

^b^ (A) dominated by (B): more lost QALYs and higher costs in total screening compared to advanced screening (ICER: -29,175 EUR)

### Effects on subpopulations

At model start, the HCV infected population consists of 69.6% (or 191,000) GEP, 29% (or 80,000) PWID, and 1.4% (or 3700) MSM. Depending on the screening strategy, the development of infection numbers differs between subgroups (Fig. 2). The general population shows similar curve progressions as total population (with infection numbers at model end between 45,000 in no screening and 1500 in total screening). As most new infections occur in the PWID subpopulation and screening (and subsequent treatment) can prevent new infections. Curve progression depends strongly on the screening strategy. While nearly constant over time in no screening (i.e. 74,000 at model end), infection numbers are decreasing, if screening is applied (up to 8700 in total screening). In the MSM subpopulation infection numbers are even increasing over time, if no screening is applied (as new infections are assumed to increase over time). With screening a (more or less) sharp decrease in infection numbers is realized in the immediate periods after screening, but is increasing later again (and even in the total screening scenario numbers at model end reach numbers from model start, while they outrun numbers at model start in basic and advanced screening).

The impact of screening on infection numbers is reflected by QALY and cost data. The loss of QALYs due to HCV infection (compared to non-infection) is reduced with more comprehensive screening strategies in all subgroups (Fig. 3). The largest QALY improvement (in absolute and relative numbers) is gained in the PWID subpopulation with reductions of 160,000 lost QALYs (or 49% from 330,000 in no screening to 170,000 in total screening) compared to a reduction of 150,000 lost QALYs in GEP (or 34% from 460,000 in no screening to 310,000 in total screening) and 4200 in MSM (or 23% from 17,500 in no screening to 13,300 in advanced screening). As for the overall population, total costs are increasing with more comprehensive screening strategies in GEP and PWIDs, but not in MSM (Fig. 4). The increase in total costs is larger in GEP (about 2.0 billion EUR) compared to PWIDs (about 0.35 billion EUR), while total costs are (nearly) unaltered in MSM.

Transition to more comprehensive screening strategies is resulting in ICERs as shown in Table 1. While ICERs are increasing sharply in GEP and MSM with more comprehensive screening strategies (amounting to 30,000 EUR per QALY for total versus advanced screening in GEP and total screening being even dominated by advanced screening in MSM), ICER is decreasing in the PWID subpopulation (to 30 EUR per QALY for total versus advanced screening). Thus, assuming a threshold of 20,000 EUR per QALY (as for the overall population) advanced screening would be recommended for application in GEP and MSM subpopulations, while total screening would be preferred for PWID subpopulation, if subgroup-specific strategies are pursued.

### Mixed strategy

Though GEP, PWID and MSM subgroups are disjunct according to subpopulation definition, they are interconnected via the fixed annual treatment capacity (25,000 treatments per year) (i.e. an increase in treatment numbers in one subpopulation induces a decrease in at least one other subgroup). Thus, efficiency of a mixed strategy derived from the cost-effective scenarios in subgroups (i.e. advanced screening in GEP and MSM, and total screening in PWIDs,) needs to be verified.

As shown in Table 1 the mixed screening strategy proves to be efficient (with ICERs of 700 EUR per QALY for mixed screening versus advanced screening, but 21,800 EUR per QALY for total screening compared to mixed screening). With mixed screening about 45,000 QALYs are gained compared to advanced screening and 0.8 billion EUR are saved compared to total screening.

### Sensitivity analysis (and scenario analysis)

**Fig. 5:**
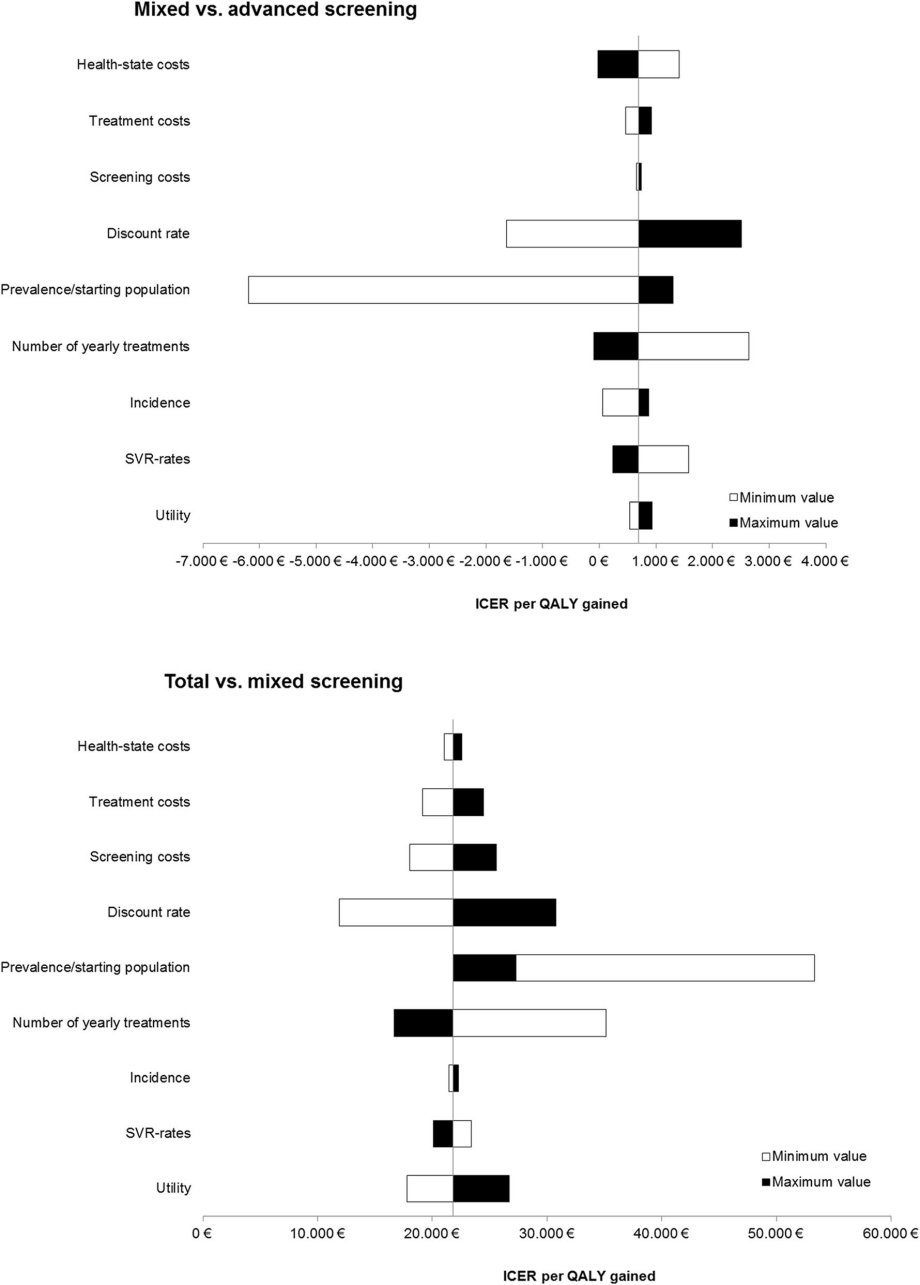
Tornado charts for incremental cost-effectiveness

In sensitivity analyses, we identified the parameters with major impact on costs per QALY results for comparing (a) mixed screening versus advanced screening and (b) total screening versus mixed screening. As shown in Fig. 5 the cost-effectiveness of mixed versus advanced screening is not affected by parameter variations (as all ICERs are less than 2600 EUR per QALY). For the comparison of total versus mixed screening, discount rate and treatment capacity have the highest impact on incremental cost-effectiveness. Expanding treatment capacities to 30,000 patients per year would decrease ICER for total versus mixed screening to 17,000 EUR per QALY. Substantial treatment price reductions (to 25,000 and 20,000 EUR) would also decrease ICER (to 19,000 and 17,500 EUR per QALY, respectively).

## Discussion

### Interpretation of the results

The aim of the present study was to analyze different screening strategies to eliminate hepatitis C virus infection in Germany until 2040 using a model that is different from all other existing models to our knowledge. Thus we applied a comprehensive approach targeting the complete German HCV-infected population. In fact, we considered all relevant target groups. GEP included the total population (except for PWIDs and MSM), in particular blood recipients before 1992, healthcare workers, and immigrants (all these subgroups were addressed via check-up 35+ based screening. PWIDs and MSM were considered as separate subpopulations as all incident cases are assumed to occur in these two subpopulations and both subgroups require a specific approach to actively participate in the screening program.

In all subpopulations, screening strategies differ in the expenses to include populations into the screening program, which is mirrored by different coverage and participation rates of target groups. In GEP, basic screening addresses the persons at risk participating in check-up 35+ (about 48% of the population 35+ at risk). Advanced screening is expanded to the general population 16+ at risk (assuming an 80% screening rate), while total screening includes the complete general population (with or without increased risk for HCV infection).

In the PWID subpopulation, basic screening is focusing on PWIDs undergoing substitution therapy, and advanced screening is, in addition, addressing PWIDs in community (with both strategies assuming participation rates of 40 to 80%), while total screening includes the total PWID subpopulation. In MSM, screening strategies just differ in the participation rate (with screening participation rates between 50 and 100%).

To address the burden of disease, GEP and PWIDs are the most relevant subgroups (as they represent nearly 99% of all HCV infections at model start in 2015), while PWIDs and MSM are the main target groups for long-term elimination of HCV infections (as preventing incident cases is crucial for long-term elimination).

Infection numbers are decreasing with all screening strategies (from 275,000 in 2015 to between 125,000 in no screening and 14,000 in total screening in 2040). During the same period, the percentage of GEP population among HCV infected population is decreasing from 70% in 2015 to between 36% (in the no screening scenario) and 11% (in the total screening scenario) in 2040, while the percentage of PWIDs is increasing from 29% to about 62% in 2040 (showing only little variation between screening strategies ranging between 59 and 65%), and the percentage of MSM is increasing from 1.4% to between 5.4% (in no screening) and 27% (in total screening). Thus, the aggregate percentage of PWIDs and MSM among the infected population increases from 30% in 2015 to between 64 and 89% in 2040, emphasizing the importance of tackling new infections for long-term elimination.

Assuming a threshold of 20,000 EUR per QALY, incremental cost-effectiveness analyses in subgroups suggest that advanced screening is efficient in GEP and MSM, while total screening is cost-effective in PWIDs. In GEP and MSM, incremental cost-effectiveness ratio is increasing with intensified screening efforts, amounting to 15,000 and 13,000 EUR per QALY for advanced compared to basic screening, respectively (while ICER for total versus advanced screening is clearly above the threshold). In contrast, ICER is decreasing with intensified screening efforts in the PWID subpopulation (with incremental cost-effectiveness of 30 EUR per QALY for total versus advanced screening).

The subgroup cost-effectiveness results are confirmed by the mixed strategy (combining advanced screening in GEP and MSM, and total screening in PWIDs) The mixed strategy proved to be efficient (with incremental cost-effectiveness ratios amounting to 700 EUR per QALY for mixed versus advanced screening, but 21,800 EUR per QALY for total compared to mixed screening). Thus, based on ICER efficiency criteria, recommendations for an overall population screening imply to focus on the risk-based screening in GEP, while in PWIDs the entire subpopulation should be tackled (and in MSM a high percentage of the subpopulation).

Though, acknowledging that it is difficult to realize the full potential of total screening, the cost-effectiveness results support efforts to include as many PWIDs as possible. In particular, total screening in PWIDs would still be cost-effective (i.e. ICER for mixed versus advanced screening would be 20,000 EUR per QALY), if social worker expenditures to include PWIDs into the screening program are expanded to about 330 h (or 43 working days) per PWID in community.

As PWIDs and MSM play a crucial role for long-term elimination (via tackling transmission), a reasonable strategy might even be to restrict HCV treatment to PWIDs and MSM in the first periods of the screen and treat program (as treatment capacities are supposed to be limited). Treatment of the general population would follow when there are free capacities (i.e. when all PWIDs and MSM accessible have been treated).

### Comparison to other studies

Several studies [40, 41] explore national prioritization strategies aiming at the overall elimination of HCV infections, but these studies lack consideration of screening measures and do not consider target group specific access strategies. Most studies addressing screening for HCV are focusing on specific target groups [42, 43] such as general population (specified as birth cohorts 1945 to 1965 or 1970, PWIDs or health care workers.

Studies on screening in the general population compare birth cohorts screening to either no screening or risk-based screening (which often corresponds to the status quo). There are several recent studies (from the period 2012–2017) conducted in various countries across Europe [44, 45], North America [46–52] and Asia [53].

Comparisons of birth cohort screening versus no screening show mixed results with incremental cost-effectiveness ratios ranging from 6000 USD to 65,000 USD per QALY gained [44, 45, 49–52]. As study comparisons show, the cost-effectiveness of screening in the general population turns out to be highly sensitive to prevalence rates in the general population (which range from 0.5 to 6% in the studies considered) [42].

Analyses of birth cohort screening versus risk-based screening are only conducted in US studies [46–48, 51], showing birth cohort screening to be cost-effective (with ICERs between 8000 and 38,000 USD). These findings are explained by high prevalence rates in the birth cohorts 1945–1970.

Besides, there are only a few studies considering the new interferon-free DAAs [45, 52, 53]. The two studies considering both, new interferon-free and other regimens, find that incremental cost-effectiveness is lower in DAAs compared to interferon-based regimens [45, 52].

Studies on screening PWIDs are from Europe [54–59] or the US [60–62]. Only one study is considering interferon-free DAAs [61, 62], while all others are from pre-DAA times. However, screening PWIDs turns out to be cost-effective (compared to no screening), as all but one studies [62] present ICERs of less than 30,000 USD per QALY, and in part PWID screening is even dominant (i.e. screening is gaining more QALYs and reducing costs compared to no screening) [58, 61]. Screening studies are conducted in different settings, addressing former PWIDs, PWIDs under opioid replacement therapy, or current PWIDs. A Dutch study [59] examines a drug user campaign where addiction care professionals provide counselling to PWIDs at their meeting venues. This intervention proves to be cost-effective (resulting in 7300 EUR per QALY).

Unlike the screening literature, our study provides a uniform analysis framework for assessing screening strategies aiming at elimination HCV infections in a nationwide approach. The framework allows considering interactions between subgroup screening decisions, examining target-specific screening approaches and deriving an optimal overall screening strategy.

In general, our findings are supported by the literature: Cost-effectiveness ratios of screening seem to be lower in PWIDs compared to the general population (which in the ‘optimum’ mixed strategy of our model results in more comprehensive screening in PWIDs compared to general population). Moreover, the literature shows that strategies to approach PWIDs in community might be cost-effective.

While the literature on screening the general population shows mixed cost-effectiveness results, it is an efficient strategy according to our model. Moreover, in contrast to findings of the US literature, risk-based screening is recommended in our model as cost-effective in the general population (compared to birth cohort screening) which might be partially due to lower prevalence rates in the German general population (born 1945 to 1965).

### Limitations

There are some limitations that have to be considered when interpreting study results. SVR-rates are based on the results from different clinical trials. Trials often overestimate efficacy data due to selected trials participants and increased health care professionals’ attention. However, recent analyses in real-life settings show comparable SVR-rates in clinical practice [63, 64]. Furthermore, efficacy data are not available for some patient subgroups (e.g. treatment-naïve versus treatment-experienced patients). Also the impact of treatment for specific populations (in particular drug users) is not based on trials data. Nevertheless, the impact on the model results might be low, as overall SVR-rates range between 90 and 100% in new DAAs. Furthermore, we stratified hepatitis C infection rates by age, but not by sex, because there is no valid data on sex-specific prevalence rates in Germany. Disregarding gender-specific prevalence rates is quite usual in the international literature on population-based screening models [65]. Moreover, we would not expect considering sex-specific prevalence rates to have significant impact on the results of our model (the most potential impact might be expected for GEP, but with similar participation rates in check-up 35+ for both sexes considering gender-specific prevalence rates does almost not affect numbers of identified HCV infected persons).

A general problem in modelling the long-term effects of screening programs is the availability and reliability of data. Extensive literature analyses have been performed to determine the best available data. Moreover, sensitivity analyses suggest that the impact of most parameters might not be critical to the cost-effectiveness assessment. However, e.g. expanding treatment capacities (from 25,000 to 30,000 patients) might affect cost-effectiveness assessment (thus including more patients into the screening program and subsequent treatment might be recommended).

Screening participation rates are also critical to the model results. We mapped variations of participation rates in the definition of the screening scenarios (thus participation is increasing from no screening via basic and advanced screening to total screening). Basic screening is modelled as a conservative scenario resting on published participation rates from Germany (e.g. participation rate of PWIDs is based on data from interferon-based regimens, while willingness to participate in screening is assumed to increase with interferon-free agents due to less adverse effects). Participation rates in advanced screening are assumed to be feasible, while participation rates in total screening are hypothetical showing the potential of screening. However, in both scenarios higher costs to include patients into the screening program are considered.

At last, we restricted the number of defined patient groups to GEP, PWIDs, and MSM, while assuming that e.g. healthcare workers and migrants (two subgroups often considered in the literature) are included in the general population. In fact, these subgroups are included in our risk-based screening approach in the GEP subpopulation (which is performed in basic and advanced screening) and thus show a higher detection rate than the “rest” of the general population reflecting their higher infection risk (though admittedly, migrants’ participation in check-up 35+ might be below-average). Nevertheless, specific screening strategies for healthcare workers and/or migrants could have been added to our screening model (though, in the end, the number of different sub-populations and screening strategies had to be restricted).

## Conclusion

- Screening is the key to an efficient decline of the HCV-infected population in Germany. Recommendation for an overall population screening is to screen the total PWID subpopulation, and to apply less comprehensive advanced screening for MSM and GEP. With new interferon-free DAAs, elimination of HCV infection in Germany seems to be feasible and cost-effective. If treatment costs decreased to 20,000 EUR per patient, even overall total screening might be a cost-effective option. This scenario is conceivable as price-reduced generics of sofosbuvir/velpatasvir and ledipasvir/sofosbuvir have been launched in the US market and might spill over to Europe soon. Critical is the participation rate of PWIDs in community as this target group is difficult to approach. Our calculations show that high resource inputs for approaching might be an efficient investment to exploit the potential of PWIDs willing to participate in the screening program.

## Supplementary information

**Additional file 1: Table S1.** Model inputs. **Table S2.** Parameters of the different screening strategies.

## Data Availability

The datasets used and/or analysed during the current study are available from the corresponding author on reasonable request.

## Abbreviations

ALT: Alanine aminotransferase
DAA: Direct acting antivirals
EUR: Euro
GEP: General population
HCV: Hepatitis C virus
HIV: Human immunodeficiency virus
ICER: Incremental cost-effectiveness ratio
MSM: HIV-positive who have sex with men
PWID: People who inject drugs
PWID-C: PWIDs in community who regularly inject drugs
PWID-S: PWIDs undergoing substitution therapy
QALY: Quality adjusted life year
RNA: Ribonucleic acid
SVR: Sustained viral response
UK: United Kingdom
US: United States
USD: US-Dollar
