# Supplementary material for: Elimination of hepatitis C virus in Germany: modelling the cost-effectiveness of HCV screening strategies
Source: BMC Infect Dis. 2019 Dec 2;19:1019. doi: 10.1186/s12879-019-4524-z (PMC6889318; doi:10.1186/s12879-019-4524-z)
Supplement: Supplementary file 1 — Additional file 1: Table S1. Model inputs. Table S2. Parameters of the different screening strategies. [file 12879_2019_4524_MOESM1_ESM.docx]

Table S1: Model inputs

| **Parameter** | **Base-Case** | **Range/ Variation** | **Source** |
| --- | --- | --- | --- |
| **Prevalence rate of HCV by age** |  |  |  |
| Age-group 1 (15-34) | 0.08% | ±25% | [1] |
| Age-group 2 (35-64) | 0.43% | ±25% | [1] |
| Age-group 3 (65+) | 0.50% | ±25% | [1] |
| **Prevalence rate of HCV by subgroup** |  |  |  |
| Overall | 0.30% |  | [2] |
| Check up 35/general population | 0.25% |  | Calculated |
| Medical personnel | 0.69% |  | [3] |
| PWIDs under substitution | 42.00% |  | [4] |
| PWID community based | 42.00% |  | [4] |
| MSM | 8.20% |  | [5, 6] |
| **Distribution of disease severity by age** |  |  |  |
| **Age-group 1 (15-34)** |  |  |  |
| F0 | 72.68% |  | [1] |
| F1 | 22.96% |  | [1] |
| F2 | 3.52% |  | [1] |
| F3 | 0.71% |  | [1] |
| F4 | 0.13% |  | [1] |
| DCC | 0.00% |  | [1] |
| HCC | 0.00% |  | [1] |
| Liver transplantation | 0.00% |  | [1] |
| Post liver transplantation | 0.00% |  | [1] |
| **Age-group 2 (35-64)** |  |  |  |
| F0 | 26.53% |  | [1] |
| F1 | 34.44% |  | [1] |
| F2 | 16.77% |  | [1] |
| F3 | 14.97% |  | [1] |
| F4 | 6.78% |  | [1] |
| DCC | 0.29% |  | [1] |
| HCC | 0.17% |  | [1] |
| Liver transplantation | 0.04% |  | [1] |
| Post liver transplantation | 0.00% |  | [1] |
| **Age-group 3 (65+)** |  |  |  |
| F0 | 4.83% |  | [1] |
| F1 | 17.60% |  | [1] |
| F2 | 15.04% |  | [1] |
| F3 | 24.02% |  | [1] |
| F4 | 34.21% |  | [1] |
| DCC | 3.25% |  | [1] |
| HCC | 0.85% |  | [1] |
| Liver transplantation | 0.21% |  | [1] |
| Post liver transplantation | 0.00% |  | [1] |
| **Distribution of disease by genotype** |  |  |  |
| Genotype 1 | 63.00% |  | [2] |
| Genotype 2 | 6.00% |  | [2] |
| Genotype 3 | 27.00% |  | [2] |
| Genotype 4-6 | 4.00% |  | [2] |
| **Incidence** |  |  |  |
| **Incident cases of HCV by subgroup** |  |  |  |
| Overall | 4,887 |  | [7] |
| PWID | 4,398 |  | Assumption: incident cases only in PWID and MSM; distribution PWID/MSM = 90/10 |
| MSM | 489 |  | Assumption: incident cases only in PWID and MSM; distribution PWID/MSM = 90/10 |
| **Transition probabilities** |  |  |  |
| F0 to F1 | 10.90% |  | [8] |
| F1 to F2 | 6.80% |  | [8] |
| F2 to F3 | 11.30% |  | [8] |
| F3 to F4 | 12.50% |  | [8] |
| F4 to DCC | 3.90% |  | [9] |
| F4 to HCC | 1.40% |  | [9] |
| F4 (SVR) to DCC | 0.33% |  | [10–13] |
| F4 (SVR) to HCC | 0.34% |  | [10–13] |
| DCC to HCC | 1.40% |  | [9] |
| DCC to liver transplant | 3.10% |  | [9] |
| DCC to death | 12.90% |  | [9] |
| HCC to death | 42.70% |  | [9] |
| Liver transplant to liver transplant | 79.00% |  | [9] |
| Liver transplant to death | 21.00% |  | [9] |
| Post liver transplant to death | 5.70% |  | [9] |
| **Mortality rate by age** |  |  |  |
| Age-group 1 (15-34) | 0.04% |  | [14] |
| Age-group 2 (35-64) | 0.37% |  | [14] |
| Age-group 3 (65+) | 2.40% |  | [14] |
| **Diagnosis rate by disease severity** |  |  |  |
| F0 | 60.00% |  | [2] |
| F1 | 60.00% |  | [2] |
| F2 | 60.00% |  | [2] |
| F3 | 60.00% |  | [2] |
| F4 | 60.00% |  | [2] |
| **Treatment efficacy (SVR) for DAAs. by genotype and disease severity** |  |  |  |
| **Genotype 1** |  |  |  |
| F0 | 98.50% | ±25% | [15] |
| F1 | 98.50% | ±25% | [15] |
| F2 | 98.50% | ±25% | [15] |
| F3 | 98.50% | ±25% | [15] |
| F4 | 99.00% | ±25% | [15] |
| **Genotype 2** |  |  |  |
| F0 | 99.00% | ±25% | [15] |
| F1 | 99.00% | ±25% | [15] |
| F2 | 99.00% | ±25% | [15] |
| F3 | 99.00% | ±25% | [15] |
| F4 | 99.00% | ±25% | [15] |
| **Genotype 3** |  |  |  |
| F0 | 98.00% | ±25% | [15] |
| F1 | 98.00% | ±25% | [15] |
| F2 | 98.00% | ±25% | [15] |
| F3 | 98.00% | ±25% | [15] |
| F4 | 93.00% | ±25% | [15] |
| **Genotype 4-6** |  |  |  |
| F0 | 99.00% | ±25% | [15] |
| F1 | 99.00% | ±25% | [15] |
| F2 | 99.00% | ±25% | [15] |
| F3 | 99.00% | ±25% | [15] |
| F4 | 99.00% | ±25% | [15] |
| **Utilities** |  |  |  |
| **General population by age** |  |  |  |
| Age-group 1 (16-34) | 0.95 |  | [16] |
| Age-group 2 (35-64) | 0.91 |  | [16] |
| Age-group 3 (65+) | 0.8 |  | [16] |
| **HCV cohort by disease severity** |  |  |  |
| F0 | 0.83 |  | [17] |
| F1 | 0.83 |  | [17] |
| F2 | 0.76 |  | [17] |
| F3 | 0.76 |  | [17] |
| F4 | 0.74 |  | [17] |
| F4 (SVR) | 0.78 |  | [18] |
| DCC | 0.72 |  | [17] |
| Liver transplantation | 0.79 |  | [17] |
| Post transplantation | 0.79 |  | Assumption |
| HCC | 0.72 |  | [17] |
| **Treatment costs** |  |  |  |
| DAA | 34,000 € | ±25% | [19, 20] |
| DAA | 25,000 € | ±25% | Assumption |
| DAA | 20,000 € | ±25% | Assumption |
| Annual price reduction of the DAA | 4,00% |  | Assumption |
| Discounting | 3,00% | 0%, 5% | [21] |
| **Proportion of relevant HCV population receiving pension/early retirement due to HCV per year** |  |  | [22–29] |
| B18 Chronic Viral Hepatitis (F0-F3) | 0.063727% |  |  |
| K74 Fibrosis and Cirrhosis of the Liver (F4-DCC) | 0.476638% |  |  |
| C22 Liver Cancer (HCC & LTx) | 5.396230% |  |  |
| **Proportion of relevant HCV population in rehabilitation due to HCV per year** |  |  | [22–29] |
| B18 Chronic Viral Hepatitis (F0-F3) | 0.112251% |  |  |
| K74 Fibrosis and Cirrhosis of the Liver (F4-DCC) | 0.485487% |  |  |
| C22 Liver Cancer (HCC & LTx) | 17.650318% |  |  |
| **Percentage of HCV cohort with rehabilitation costs** |  |  | [22–29] |
| B18 Chronic Viral Hepatitis (F0-F3) | 0.11% |  |  |
| K74 Fibrosis and Cirrhosis of the Liver (F4-DCC) | 0.49% |  |  |
| C22 Liver Cancer (HCC & LTx) | 17.65% |  |  |
| **Precaution and rehabilitation costs** |  |  |  |
| B18 Chronic Viral Hepatitis (F0-F3) | 2,928 € |  | [17] |
| K74 Fibrosis and Cirrhosis of the Liver (F4-DCC) | 2,729 € |  | [17] |
| C22 Liver Cancer (HCC & LTx) | 2,686 € |  | [17] |
| **Direct costs by disease severity** |  |  |  |
| F0 | 107 € | ±25% | [30] |
| F1 | 174 € | ±25% | [30] |
| F2 | 391 € | ±25% | [30] |
| F3 | 889 € | ±25% | [30] |
| F4 | 2,232 € | ±25% | [30] |
| F4 (SVR) | 2,232 € | ±25% | [30] |
| DCC | 9,767 € | ±25% | [31, 32] |
| HCC | 26,726 € | ±25% | [31, 32] |
| Liver transplantation | 148,046 € | ±25% | [33] |
| Post transplantation | 25,042 € | ±25% | [33] |
| **Indirect costs** |  |  |  |
| **Labour Force Participation** |  |  |  |
| General population | 54.30% |  | [34] |
| Average labour cost per year | 38,700 € |  | [34] |
| **Labour Force Participation by disease severity** |  |  |  |
| F0 | 54.30% |  | [35] |
| F1 | 54.30% |  | [35] |
| F2 | 54.30% |  | [35] |
| F3 | 54.30% |  | [35] |
| F4 | 54.30% |  | [35] |
| **Productivity loss per employed due to HCV** |  |  |  |
| F0-F3 patients | 5.771 € |  | [36] |
| F4 patients | 5.771 € |  | [36] |
| **Screening** |  |  |  |
| **Size of population** |  |  |  |
| Population 35+ | 44,972,765 |  | [37] |
| Check up 35 | 21,627,403 |  | [38] |
| Medical personnel | 2,113,000 |  | [34] |
| PWIDs under sub. | 77,500 |  | [39] |
| PWID community based | 112,500 |  | [39] |
| MSM | 51,000 |  | [4] |
| **Medical fees** |  |  |  |
| GPT | 0.25 € |  | [40] |
| Anti-HCV | 9.80 € |  | [40] |
| HCV PCR quantitative | 89.50 € |  | [40] |
| EBM Honorary consultancy | 21.08 € |  | [40] |
| Blood examination | 0.40 € |  | [40] |

Table S2: Parameters of the different screening strategies

|  |  |  |  |  |
| --- | --- | --- | --- | --- |
|  | **General population** | **PWID** | | **MSM** |
|  |  | **Community** | **Substitution** |  |
| **Source** | [37, 41–43] | [4, 39] | | [5, 44] |
| **Basic Screening** | | | | |
| Population Size | 69,414,404 | 112,500 | 77,500 | 51,000 |
| Potential Check-Up 35 Population | 44,972,765 |  |  |  |
| Check-Up Participation Rate | 48% |  |  |  |
| Check-up participants | 21,627,403 |  |  |  |
| HCV RNA prevalence rate | 0.28% | 42.00% | 42.00% | 7.30% |
| Infected persons (total) | 191,477 | 47250 | 32550 | 3723 |
| Patients already detected | 92,359 | 33548 | 23111 | 2643 |
| Undetected patients | 99,118 | 13703 | 9440 | 1080 |
| Awareness rate | 48% | 71% | 71% | 71% |
| Anti-HCV screening rate | **53%** | **0%** | **50%** | **50%** |
| Anti-HCV Prevalence | 0.41% | 66% | 66% | 8.10% |
| HCV-RNA positive patients among anti-HCV-positive | 66.67% | 64% | 64% | 90% |
| Screened for Anti-HCV | 11,376,014 | 0 | 38,750 | 25,500 |
| Screened for HCV-RNA | 47,070 | 0 | 25,575 | 2,066 |
| HCV-RNA positive (eligible for treatment) | 31,380 | 0 | 16,275 | 1,862 |
| Number of newly identified HCV-Infected | 26,250 |  |  |  |
| **Advanced Screening** |  |  |  |  |
| Population Size | 69,414,404 | 112,500 | 77,500 | 51,000 |
| Potential Screening Population | 69,414,404 |  |  |  |
| Check-Up Participation Rate | 80% |  |  |  |
| Check-up participants | 55,531,523 |  |  |  |
| HCV RNA prevalence rate | 0.28% | 42.00% | 42.00% | 7.30% |
| Infected persons (total) | 191,477 | 47250 | 32550 | 3723 |
| Patients already detected | 92,359 | 33548 | 23111 | 2643 |
| Undetected patients | 99,118 | 13703 | 9440 | 1080 |
| Awareness rate | 48% | 71% | 71% | 71% |
| Anti-HCV screening rate | **53%** | **40%** | **80%** | **80%** |
| Anti-HCV Prevalence | 0.41% | 66% | 66% | 8.10% |
| HCV-RNA positive patients among anti-HCV-positive | 66.67% | 64% | 64% | 90% |
| Screened for Anti-HCV | 29,209,581 | 45,000 | 62,000 | 40,800 |
| Screened for HCV-RNA | 120,860 | 29,700 | 40,920 | 3,305 |
| HCV-RNA positive (eligible for treatment) | 80,574 | 18,900 | 26,040 | 2,978 |
| Number of newly identified HCV-Infected | 67,400 |  |  |  |
| **Total Screening** |  |  |  |  |
| Population Size | 69,414,404 | 112,500 | 77,500 | 51,000 |
| HCV RNA prevalence rate | 0.28% | 42.00% | 42.00% | 7.30% |
| Infected persons (total) | 191,477 | 47250 | 32550 | 3723 |
| Patients already detected | 92,359 | 33548 | 23111 | 2643 |
| Undetected patients | 99,118 | 13703 | 9440 | 1080 |
| Awareness rate | 48% | 71% | 71% | 71% |
| Anti-HCV screening rate | **100%** | **100%** | **100%** | **100%** |
| Anti-HCV Prevalence | 0.41% | 66% | 66% | 8.10% |
| HCV-RNA positive patients among anti-HCV-positive | 66.67% | 64% | 64% | 90% |
| Screened for Anti-HCV | 69,414,404 | 112,500 | 77,500 | 51,000 |
| Screened for HCV-RNA | 287,216 | 74,250 | 51,150 | 4,131 |
| HCV-RNA positive (eligible for treatment) | 191,477 | 47,250 | 32,550 | 3,723 |
| Number of newly identified HCV-Infected | 99,118 |  |  |  |

References

[1] Razavi H, Waked I, Sarrazin C, Myers RP, Idilman R, Calinas F et al. The present and future disease burden of hepatitis C virus (HCV) infection with today's treatment paradigm. J Viral Hepat 2014;21(Suppl. 2):34–59.

[2] Bruggmann P, Berg T, Øvrehus ALH, Moreno C., Brandão Mello CE, Roudot-Thoraval F et al. Historical epidemiology of hepatitis C virus (HCV) in selected countries. Journal of Viral Hepatitis 2014;21 Suppl 1:5–33.

[3] Westermann C, Peters C, Lisiak B, Lamberti M, Nienhaus A. The prevalence of hepatitis C among healthcare workers: a systematic review and meta-analysis. Occupational and environmental medicine 2015;72(12):880–8.

[4] Robert Koch Institut. HIV, Hepatitis B und C bei injizierenden Drogengebrauchenden in Deutschland – Ergebnisse der DRUCK-Studie des RKI; 2015.

[5] Jordan AE, Perlman DC, Neurer J, Smith DJ, Des Jarlais, Don C, Hagan H. Prevalence of hepatitis C virus infection among HIV+ men who have sex with men: a systematic review and meta-analysis. International journal of STD & AIDS 2017;28(2):145–59.

[6] Martin NK, Thornton A, Hickman M, Sabin C, Nelson M, Cooke GS et al. Can Hepatitis C Virus (HCV) Direct-Acting Antiviral Treatment as Prevention Reverse the HCV Epidemic Among Men Who Have Sex With Men in the United Kingdom? Epidemiological and Modeling Insights. Clinical Infectious Diseases 2016;62(9):1072–80.

[7] Robert Koch Institut. Zur Situation bei wichtigen Infektionskrankheiten in Deutschland - Hepatitis C im Jahr 2015; 2016.

[8] Thein H, Yi Q, Dore GJ, Krahn MD. Estimation of stage-specific fibrosis progression rates in chronic hepatitis C virus infection: a meta-analysis and meta-regression. Hepatology 2008;48(2):418–31.

[9] Lidgren M, Hollander A, Weiland O, Jönsson B. Productivity improvements in hepatitis C treatment: impact on efficacy, cost, cost-effectiveness and quality of life. Scandinavian journal of gastroenterology 2007;42(7):867–77.

[10] Leidner AJ, Chesson HW, Xu F, Ward JW, Spradling PR, Holmberg SD. Cost-effectiveness of hepatitis C treatment for patients in early stages of liver disease. Hepatology 2015;61(6):1860–9.

[11] Younossi ZM, Park H, Saab S, Ahmed A, Dieterich D, Gordon SC. Cost-effectiveness of all-oral ledipasvir/sofosbuvir regimens in?patients with chronic hepatitis C virus genotype 1 infection. Aliment Pharmacol Ther 2015;41(6):544–63.

[12] Pfeil AM, Reich O, Guerra IM, Cure S, Negro F, M?llhaupt B et al. Cost-Effectiveness Analysis of Sofosbuvir Compared to Current Standard Treatment in Swiss Patients with Chronic Hepatitis C. PLoS ONE 2015;10(5):e0126984.

[13] Cure S, Guerra I, Dusheiko G. Cost-effectiveness of sofosbuvir for the treatment of chronic hepatitis C-infected patients. J Viral Hepat 2015;22(11):882–9.

[14] Federal Statistical Office. Allgemeine Sterbetafel 2010/2012; 2015.

[15] AASLD/IDSA HCV Guidance Panel. Hepatitis C guidance: AASLD-IDSA recommendations for testing, managing, and treating adults infected with hepatitis C virus. Hepatology 2015;62(3):932–54.

[16] Szende A, Janssen B, Cabases J. Self-Reported Population Health: An International Perspective based on EQ-5D. Dordrecht: Springer Netherlands; 2014.

[17] Siebert U, Ravens-Sieberer U, Greiner W, Sroczynski G, Wong JB, Kuntz KM et al. Performance of different utility assessment methods in chronic hepatitis C patients. In: Kind P, Macran S, editor. Proceedings of the 19th Plenary Meeting of the EuroQol Group 13th-14th September 2002 Discussion Papers. York: UK Centre for Health Economics; 2003, p. 175–184.

[18] Wright NMJ, Tompkins CNE. A review of the evidence for the effectiveness of primary prevention interventions for hepatitis C among injecting drug users. Harm Reduct J 2006;3(1):27.

[19] LAUER-FISCHER GmbH. Lauer-Taxe Online/German Drug Directory; 2017.

[20] Krüger K, Krauth C, Rossol S, Mauss S, Boeker KHW, Müller T, Klinker H, Pathil A, Heyne R, Stahmeyer JT. Outcomes and costs of treating hepatitis C patients with second-generation direct-acting antivirals: results from the German Hepatitis C-registry. European Journal of Gastroenterology & Hepatology;2018.

[21] Institut für Qualität und Wirtschaftlichkeit im Gesundheitswesen. Allgemeine Methoden zur Bewertung von Verhältnissen zwischen Nutzen und Kosten; 2015.

[22] Gesundheitsberichterstattung des Bundes. Diagnosedaten der Vorsorge- oder Rehaeinrichtungen mit mehr als 100 Betten. [June 30, 2017]; Available from: www.gbe.bund.de.

[23] Gesundheitsberichterstattung des Bundes. Durchschnittliches Zugangsalter bei Renten wegen verminderter Erwerbsfähigkeit in der Gesetzlichen Rentenversicherung; Available from: www.gbe.bund.de.

[24] Gesundheitsberichterstattung des Bundes. Rentenzugänge wegen verminderter Erwerbsfähigkeit in der Gesetzlichen Rentenversicherung im Laufe des Berichtsjahres. [June 30, 2017]; Available from: www.gbe.bund.de.

[25] Robert Koch Institut. Krebs in Deutschland. [June 30, 2017]; Available from: http://www.krebsdaten.de/Krebs/DE/Content/Publikationen/Krebs_in_Deutschland.

[26] Martel C de, Maucort-Boulch D, Plummer M, Franceschi S. World-wide relative contribution of hepatitis B and C viruses in hepatocellular carcinoma. Hepatology 2015;62(4):1190–200.

[27] Sivanathan V, Kittner J, Sprinzl M, Weinmann A, Koch S, Wiltink J et al. Ätiologie und Komplikationen der Leberzirrhose: Daten eines deutschen Zentrums. Dtsch med Wochenschr 2014;139(36):1758–62.

[28] Deutsche Rentenversicherung Bund. Reha-Bericht 2015; 2015.

[29] Deutsche Rentenversicherung Bund. Rentenversicherung in Zahlen 2015; 2015.

[30] Müllhaupt B, Bruggmann P, Bihl F, Blach S, Lavanchy D, Razavi H et al. Modeling the Health and Economic Burden of Hepatitis C Virus in Switzerland. PloS one 2015;10(6):e0125214.

[31] Siebert U, Sroczynski G, Rossol S, Wasem J, Ravens-Sieberer U, Kurth BM et al. Cost effectiveness of peginterferon alpha-2b plus ribavirin versus interferon alpha-2b plus ribavirin for initial treatment of chronic hepatitis C. Gut 2003;52(3):425–32.

[32] Wasem J, Sroczynski G, Aidelsburger P, Buchberger B, Hessel F, Conrads-Frank A et al. Gesundheitsökonomische Aspekte chronischer Infektionskrankheiten am Beispiel der chronischen Hepatitis C. Bundesgesundheitsbl - Gesundheitsforsch - Gesundheitsschutz 2006;49(1):57–63.

[33] Harries L, Schrem H, Stahmeyer JT, Krauth C, Amelung VE. High resource utilization in liver transplantation-how strongly differ costs between the care sectors and what are the main cost drivers?: a retrospective study. Transplant international official journal of the European Society for Organ Transplantation 2017;30(6):621–37.

[34] Federal Statistical Office. Statistisches Jahrbuch: Deutschland und Internationales; 2016.

[35] Huppe D, Zehnter E, Mauss S, B?ker K, Lutz T, Racky S et al. Epidemiologie der chronischen Hepatitis C in Deutschland - Eine Analyse von 10 326 Hepatitis-C-Virus-Infizierten aus Schwerpunktpraxen und -ambulanzen. Z Gastroenterol 2008;46(1):34–44.

[36] Vietri J, Prajapati G, El Khoury, Antoine C. The burden of hepatitis C in Europe from the patients’ perspective: a survey in 5 countries. BMC Gastroenterol 2013;13(1):107.

[37] Statistisches Bundesamt. Zensus 2011. [June 30, 2017]; Available from: https://www.destatis.de/DE/Methoden/Zensus_/Zensus.html.

[38] Gesundheitsberichterstattung des Bundes. Indikator 7.17 des Indikatorensatzes der GBE der Länder: Teilnahme am gesetzlichen Gesundheits-Check-up. [June 30, 2017]; Available from: www.gbe.bund.de.

[39] Deutsche Beobachtungstelle für Drogen und Drogensucht. REITOX-Bericht 2015. [June 30, 2017]; Available from: http://www.dbdd.de/.

[40] Kassenärztliche Bundesvereinigung. Einheitlicher Bewertungsmaßstab. [June 30, 2017]; Available from: http://www.kbv.de/html/ebm.php.

[41] Wolffram I, Petroff D, Bätz O, Jedrysiak K, Kramer J, Tenckhoff H et al. Prevalence of elevated ALT values, HBsAg, and anti-HCV in the primary care setting and evaluation of guideline defined hepatitis risk scenarios. Journal of Hepatology 2015;62(6):1256–64.

[42] The Federal Health Monitoring System. Utilization of Statutory Health Ckeck-up. [June 30, 2017]; Available from: www.gbe.de.

[43] Poethko-Müller C, Zimmermann R, Hamouda O, Faber M, Stark K, Ross RS et al. Die Seroepidemiologie der Hepatitis A, B und C in Deutschland. Bundesgesundheitsbl. 2013;56(5-6):707–15.

[44] Robert Koch Institut. Weiterführende Analysen zur HIV-Inzidenz- und -Prävalenzschätzung 2012; 2013.
